# Cardiovascular MR function and coronaries: CMR 15 minute express

**DOI:** 10.1186/1532-429X-15-S1-T11

**Published:** 2013-01-30

**Authors:** Mary P Watkins, Todd A Williams, Shelton D Caruthers, Samuel A Wickline

**Affiliations:** 1 Washington University, St. Louis, MO, USA

## Background

CMR imaging has become a recognized gold-standard diagnostic technique for evaluating cardiovascular function, but has limited success in developing coronary imaging as a screening tool. With the faster image acquisition and reconstruction schemes, CMR can now be utilized as a non-invasive and robust screening modality of cardiovascular function and coronary disease in only 15 minutes of scanning. The 15 Minute Express Screening will enable further CMR imaging while maintaining a reasonable study length.

To develop a high image-quality, repeatable, robust, and rapid clinical CMR diagnostic screening workflow, shorter than 15min, for cardiovascular function and coronary arteries.

## Methods

**Figure 1:**
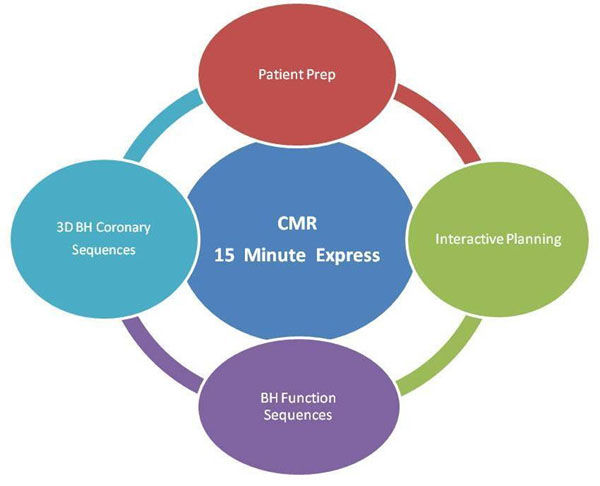
CMR 15 Minute Express – average completion time 15 min and 25 sec

To obtain a CMR Express several steps must be completed. We imaged 8 volunteers, with a five-element cardiac synergy coil, on a clinical 1.5T scanner (Philips Achieva Release 2.5.3, Best, Netherlands), with a systematic approach shown in Figure 1. All sequences were acquired as breath hold (BH) balanced-TFE with SENSE acceleration.

1. Patient preparation outside of the magnet: The first step is to prepare the patient outside of the magnet, including skin preparation, VCG placement, and verbal BH instructions. Coaching is essential for BH success. Finally, place the patient, head-first and supine, in the magnet bore.

2. Use interactive planning for function views: All cine and coronary sequences should be pre-loaded and the geometry parameter set to reference the appropriate cine views to be defined interactively. The first view planned is a VLA (vertical long axis), followed by HLA (horizontal long axis), SA (short axis), and LVOT (left vertical outflow track) views all planned interactively, storing geometries in the respective parameter.

3. Pre-loaded sequences are acquired to complete the study: Next, based on the previously-stored geometries, cine sequences are started and a series of BHs begins. Single slice cine images with at least 30 heart phases are obtained of the HLA, LVOT, and VLA. Multiple slice stack cine images are obtained of the short axis (SA) of ventricles. 3D CAIs are planned from basal SA. The average BH was 10-20sec.

## Results

**Figure 2:**
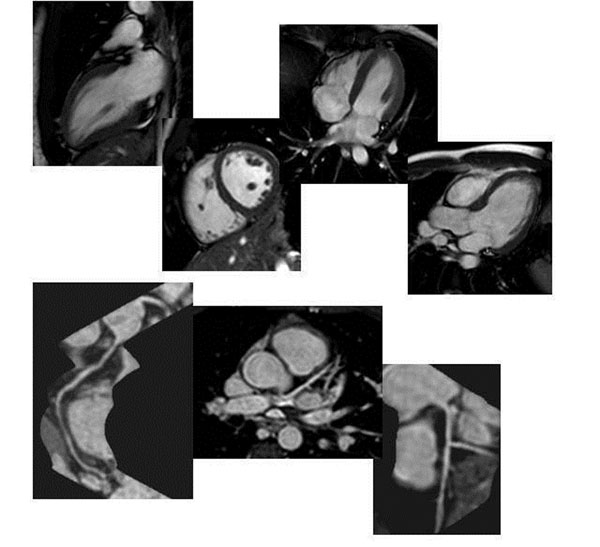
Function Cine (VLA, SA, HLA, LVOT-top row) and Coronary Images (reformatted RCA, 3D LCA, reformatted LCA-bottom row)

The studies were well tolerated by all 8 volunteers with an average age of 48 years. The most significant decreases in study duration came from the interactive planning and BH CAIs portions, which trimmed about 15-20min (dependent upon patient HR) compared to free-breathing techniques. See Figure 2.

## Conclusions

The use of a systematic study protocol decreases patient time in the scanner making a rapid screening of cardiovascular function and coronaries possible.

## Funding

Washington University

